# Retrobulbar Hemodynamics and Visual Field Progression in Normal Tension Glaucoma: A Long-Term Follow-Up Study

**DOI:** 10.1155/2015/158097

**Published:** 2015-10-18

**Authors:** D. Kuerten, M. Fuest, E. C. Koch, A. Koutsonas, N. Plange

**Affiliations:** Department of Ophthalmology, RWTH Aachen University, Pauwelsstraße 30, 52072 Aachen, Germany

## Abstract

*Purpose*. Vascular risk factors are important factors in the pathogenesis of glaucoma. The purpose of this research was to investigate retrobulbar hemodynamics and visual field progression in patients with normal tension glaucoma (NTG).* Patients and Methods*. 31 eyes of 16 patients with NTG were included in a retrospective long-term follow-up study. Colour Doppler imaging was performed at baseline to determine various CDI parameters in the different retrobulbar vessels. The rate of visual field progression was determined using the Visual Field Index (VFI) progression rate per year (in %). To be included in the analysis, patients had at least 4 visual field examinations with a follow-up of at least 2 years.* Results*. Mean follow-up was 7.6 ± 4.1 years with an average of 10 ± 5 visual field tests. The mean MD (mean defect) at baseline was −7.61 ± 7.49 dB. The overall VFI progression was −1.14 ± 1.40% per year. A statistical significant correlation between VFI progression and the RI of the NPCA and PSV of the CRA was found.* Conclusion*. Long-term visual field progression may be linked to impaired retrobulbar hemodynamics in patients with NTG only to a limited degree. Interpretation of the data for an individual patient seems to be limited due to the variability of parameters.

## 1. Introduction

Glaucoma, defined as a progressive optic neuropathy, characterized by optic disc excavation and typical visual field loss, encloses various mechanical and vasogenic mechanisms in its pathogenetic concepts [1–4]. According to the WHO, it is the most frequent cause of preventable irreversible blindness [5, 6]. Colour Doppler imaging (CDI), a noninvasive ultrasound technique, allows easily assessing retrobulbar blood flow parameters, although a marked user dependency is apparent [7, 8]. Various studies showed that CDI parameters differ in patients suffering from primary open-angle glaucoma (POAG) and normal tension glaucoma (NTG) [9–12] compared to controls. The diagnostic precision however is limited due to a large overlap between groups. Previous studies using CDI showed that decreased flow velocities especially in the central retinal artery and the short posterior ciliary arteries and higher resistive indices are a reproducible finding in glaucoma suggesting decreased blood flow to the optic nerve. A vicious circle of decreased flow velocities, increased peripheral vascular resistance, and capillary dropout as seen in angiographic studies was suggested [13]. In addition, alteration in flow velocities of retrobulbar vessels is linked to functional defects in glaucoma [14–16]. Interocular asymmetric visual field defects were found to show interocular differences in CDI measurements [14].

Only few studies with limited follow-up were performed to investigate the relation of CDI parameters with future progression in glaucoma.

Studies with varying participant numbers have found that varying CDI parameters are correlated with visual field defect progression in POAG and NTG. Nevertheless, the data is still inconclusive.

Therefore, we decided to monitor the visual field defect progression of patients with NTG in a retrospective long-term follow-up study.

## 2. Patients and Methods

Thirty-one eyes of 16 patients (8 females and 8 males, mean age 60.6 ± 12 years) with NTG were included in this retrospective study that fulfilled the inclusion criteria.

Patients with NTG had glaucomatous excavation of the optic disc and a glaucomatous visual field defect as defined by the European Glaucoma Society. The diagnostic criteria for glaucomatous visual field loss are as follows. Field loss was considered significant when (a) glaucoma hemifield test was abnormal, (b) 3 points are confirmed with *p* < 0.05 probability of being normal (one of which should have *p* < 0.01), not contiguous with the blind spot, or (c) corrected pattern standard deviation (CPSD) was abnormal with *p* < 0.05. All parameters were confirmed on two consecutive visual fields performed with Humphrey Field Analyzer. All patients with glaucomatous visual field loss underwent diurnal curves of IOP measurements (Goldmann applanation tonometry) at 8.00 h, 12.00 h, 16.00 h, 20.00 h, and 24.00 h without any topical or systemic IOP-lowering medication. In patients with NTG diagnosis was confirmed by readings of IOP never above 21 mmHg.

All patients had no other serious eye diseases (e.g., age-related macular degeneration, diabetic retinopathy, and vascular occlusive diseases) and no intraocular surgeries (i.e., cataract surgery and glaucoma surgery) were performed during the observation period.

All CDI measurements were acquired in the context of other prospective clinical trials [12–14, 25–27] performed during 2001 and 2002. The inclusion criteria for this study were as follows: no intraocular surgery (e.g., cataract or glaucoma surgery) during the follow-up period. The antiglaucomatous medication was documented at the beginning and the end of the follow-up period. The average used number of different topical medications at baseline was 0.6 with a maximum of 2 different drugs (dorzolamide, timolol, brimonidine, latanoprost, and combinations). No systemic antiglaucomatous medication was taken over the course of the follow-up period. However, in all patients, antiglaucomatous therapy was altered during the follow-up period at least once.

All patients had a minimal follow-up period of 2 years and a total of at least 4 visual field examinations.

Retrobulbar blood flow velocities were acquired by means of CDI (Siemens Sonoline Sienna, http://www.healthcare.siemens.de/ultrasound, Germany) using a 7.5 MHz linear phased-array transducer. The measurements have been conducted by the same experienced investigator (NP) and the method was previously described in detail [28, 29]. CDI enables a noninvasive measurement of the blood velocity in the ophthalmic artery (OA), the central retinal artery (CRA), and nasal and temporal posterior ciliary arteries (PCAs). The peak systolic velocity (PSV) and end-diastolic velocity (EDV) were recorded for each artery. All measurements were acquired in the supine position. The resistive index was calculated (RI: PSV − EDV/PSV).

Systemic systolic blood pressure (SBP) and diastolic blood pressure (DBP) and heart rate were recorded in the supine position before CDI measurements after a rest of 10 minutes. Mean arterial pressure (MAP) was calculated and used for analysis (MAP = DBP + 1/3 SBP).

Over the follow-up period of at least 2 years at least 4 visual field examinations (24/2 SITA) (Humphrey Field Analyzer, Carl Zeiss Meditec, Germany) were performed by each subject. To be included in the analysis, the rate of false negative answers had to be <30% and that of false positive answers <20%.

The rate of visual field progression was determined using the Visual Field Index (VFI) progression rate per year (in %, Forum Glaucoma Workplace, Humphrey Field Analyzer, Carl Zeiss Meditec, Germany).

The Visual Field Index was first introduced by Bengtsson and Heijl [30] to facilitate progression analysis in glaucoma and lessen the influence of cataract and cataract surgery on the visual field analysis via MD. For each location the measured sensitivity was expressed as a percentage of the sensitivity expected in a healthy observer matched in age. The VFI is then calculated as the weighted mean of all locations with pattern deviation probability outside normal limits (<5%).

Casas-Llera et al. [31] reported that the VFI progression rate is a reliable tool to detect progression in patients with a greater number of visual field tests and a longer follow-up time and appears to be more accurate than MD analysis for determining the rate of visual field defect progression.

Age, baseline mean deviation (MD) of the Humphrey perimeter, follow-up period, number of field examinations, intraocular pressure (IOP) at baseline, MAP, and the CDI parameters of the OA, CRA, and nasal and temporal PCAs were included in a multiple regression model to the visual field progression rate.

## 3. Statistical Analysis

A simple multiple regression analysis was used in this pilot study. Fisher's transformation was used to find statistically significant correlations between the individual parameters. *p* values were set at 0.05 to be considered statistically significant.

## 4. Results

Mean follow-up was 7.6 ± 4.1 years (range 2–14 years) with an average of 10 ± 5 visual field tests (range 4–21). The mean IOP at baseline was 15.4 ± 2.0 mmHg. The baseline MD was −7.61 ± 7.49 dB. Mean age at baseline was 60.6 ± 11.9 years. The overall VFI progression was −1.14 ± 1.40% per year (range −6.1 ± 0.7).

The baseline CDI parameters of all vessels are shown in Table 1.

A statistical significant correlation between VFI progression and the RI of the NPCA (*r* = −0.43, *p* = 0.01) and PSV of the CRA (*r* = 0.37, *p* = 0.043) was found.

**Figure 1:**
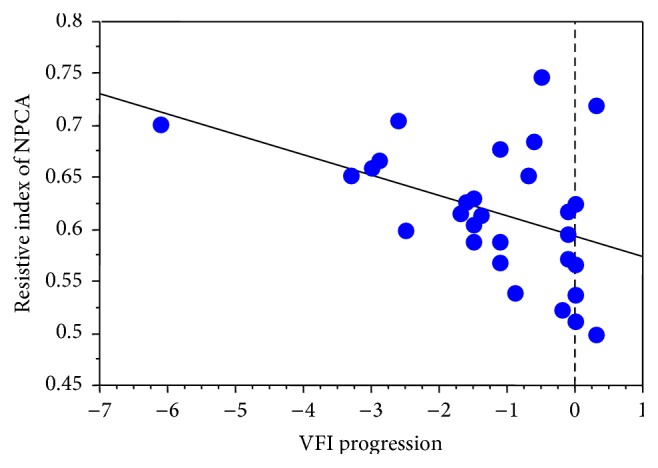
Scatterplot with regression. *x*-axis: VFI progression, *y*-axis: RI of the NPCA.

**Figure 2:**
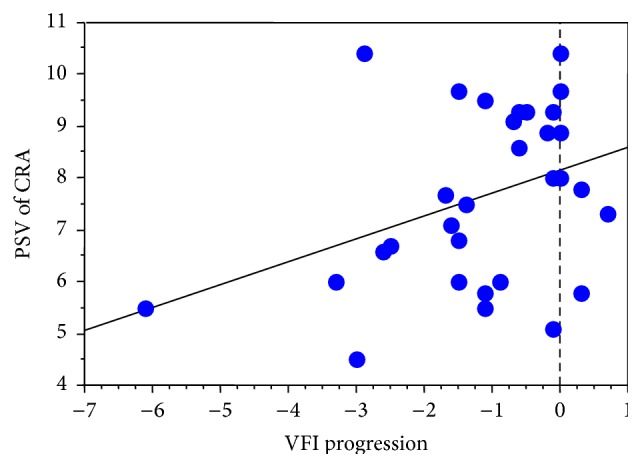
Scatterplot with regression. *x*-axis: VFI progression, *y*-axis: PSV of the CRA.

Refer to Figures 1 and 2 for regression analysis.

No significant correlation was found for the blood flow parameters of the OA and the TPCA. A significant correlation was found with baseline MD (*r* = 0.44, *p* = 0.01).

No statistical significant correlation was found for VFI progression and age, baseline IOP, number of visual field examinations, mean arterial pressure, and follow-up period, respectively. See Table 2 for details.

When removing one outlier (visual field progression index −6.1 dB per year) the correlation of both parameters was no longer significant (CRA PSV *r* = 0.31, *p* = 0.11; RI of the NPCA *r* = −0.36, *p* = 0.056).

## 5. Discussion

Ocular blood flow is an important factor in glaucomatous pathogenesis. It has been postulated that retrobulbar perfusion changes could be noted prior to glaucomatous damage [32]. Further studies found that patients with progressive glaucomatous damage and patients suffering from NTG show altered retrobulbar hemodynamics [33]. Interestingly, CDI measurements showed no significant differences in patients suffering from NTG in comparison to POAG [11] suggesting that the decreased ocular blood flow may be an important factor in all patients with open-angle glaucoma.

CDI measurements have provided an important insight into the pathogenesis of glaucoma [12–14, 27]. However, the CDI measurement's accuracy and reproducibility are variable [29]; particularly, measurements of the SPCAs show a great variability [29, 34, 35]. Ehrlich et al. [36] reported rather poor intra- and interobserver ICCs especially for the SPCAs, highlighting the methods' user dependency and overall variability as a flaw.

Overall progression of visual field defects in glaucoma is still not completely understood today. Various studies have shown that deterioration of visual field occurred despite a therapeutic IOP reduction [37, 38]. We were capable of showing that retrobulbar perfusion parameters were improved in the long term after trabeculectomy [25]. The visual field defects did not progress in these patients although a significant increase in the IOP was recorded after surgery. These and other results highlight the very likely critical involvement of ocular blood flow amongst other factors in the progression of glaucomatous visual field defects.

We were able to show a statistically significant correlation between the RI in NPCA and VFI progression, whereas the RI values in the other retrobulbar vessels showed no statistically significant correlation to VFI progression. Furthermore, a significant correlation between VFI progression and PSV in the CRA was recorded. These significant correlations did not reach statistical significance after removing one outlier (patient with visual field progression of 6.1 dB per year). In addition, the correlation between PSV in the OA and VFI progression slightly failed to reach statistical significance (*p* = 0.06) in our patients suffering from NTG after removing the outlier. However, these findings might be restricted by the small sample size and heterogeneity in the manifestation of the disease in the study population. In addition, the variability of the follow-up period, as well as of the number of visual field tests, might confound the results, although the visual field progression index (in dB per year) aims to be comparable between patients. Furthermore, the baseline MD showed a statistically significant correlation with VFI progression. Like in previous published work [19], we did not find any significant correlations between age, systemic blood pressure, and number of visual field examinations with visual field deterioration.

Please refer to Table 3 for an overlook on the existing literature on CDI measurements and visual field progression in glaucoma.

As measuring the resistive index of the retrobulbar vessels by means of CDI tends to provide the most important parameter in the assessment of disturbed retrobulbar blood flow in glaucoma [39], it is not that surprising that higher RI values are often recorded in patients suffering from glaucoma and RI values can often be attributed to the diseases' progression.

Some authors tried to determine a RI threshold of the OA in patients suffering from POAG [21, 22]. A RI value > 0.75 appears to be associated with a higher risk of visual field deterioration. Furthermore, decreased blood flow velocities (PSV and EDV), especially of the central retinal artery, were often found in patients showing a progressive disease; however, the vessels with statistically significant correlations to visual field defect progression vary amongst the studies. This can possibly be attributed to the variability in the measurement technique as mentioned above and the often rather small samples sizes in the different studies.

The cause for these discrepancies in the literature is not yet clearly identified. It is questionable whether the technique of CDI with its flaws is the sole cause for these varying results. Maybe further factors are influencing the different results. For example, the studies are not standardized in the application of topical medication, which is known to influence retrobulbar perfusion [40]. A potential bias in our study among other studies is the application of topical antiglaucoma treatment over the course of the observation period, as well as any systemic medication affecting blood flow. Furthermore, we are still unable to provide means to ensure and safely monitor the patients' compliance to a proposed therapy. Therefore, IOP changes and IOP peaks can possibly be a confounding factor and were not yet included in any of these studies. These factors, together with the variability of ocular blood flow itself, are important limitations to the application of ocular blood flow determination in clinical practice via CDI.

Furthermore, the observation period in many studies appears to be insufficient to safely identify all patients with progressive visual field defects.

Overall our tools to identify progression of the disease are somewhat rough and limited and therefore the beginning of progression can easily be missed especially if the observation period only spans up to two years. As shown in Table 3 there is no general consensus about the best diagnostic tool to identify progression in glaucoma; most authors prefer visual field changes; others prefer optic disc changes via morphometric techniques. Nevertheless, these changes require time and are irreversible; therefore, further factors have to be investigated to identify vulnerable patients and prevent disease progression.

In conclusion, CDI measurements seem not to be able to provide a safe evaluation for an individual patient regarding the risk of progression. It is not possible to determine the obvious best parameter that is correlated with glaucoma progression due to the high variability of results found in previously published studies and in our studies. Nevertheless, retrobulbar hemodynamics and ocular perfusion appear to play a major role among other factors, some of which are not clearly defined today, in glaucoma progression. Therefore, blood flow measurements should not be overlooked in the search for better means to identify patients at risk.

## Figures and Tables

**Table 1:** CDI measurements in patients with NTG (*n* = 31) of the ophthalmic artery (OA), central retinal artery (CRA), and nasal and temporal posterior ciliary arteries (PCAs). The peak systolic velocities (PSV), end-diastolic velocities (EDV), and resistive indices (RI) are displayed (mean and SD).

	OA	CRA	Nasal PCA	Temporal PCA
PSV	34.9 ± 8.25	7.64 ± 1.69	7.77 ± 1.85	7.79 ± 1.82
EDV	8.6 ± 3.1	2.57 ± 0.67	2.94 ± 0.71	2.9 ± 0.69
RI	0.75 ± 0.06	0.65 ± 0.08	0.62 ± 0.06	0.62 ± 0.06

PSV: peak systolic velocity.

EDV: end-diastolic velocity.

RI: resistive index.

OA: ophthalmic artery.

CRA: central retinal artery.

NPCA: nasal posterior ciliary artery.

TPCA: temporal posterior ciliary artery.

**Table 2:** Correlation between VFI progression and other parameters including CDI parameters using Fisher's transformation (*p* < 0.05 was perceived as a statistical significant correlation). When removing one outlier (visual field progression index −6.1 dB per year) the correlation of the PSV of the CRA (*r* = 0.31, *p* = 0.11) and of RI of the NPCA (*r* = −0.36, *p* = 0.056) was no longer significant.

Correlation between VFI progression and other parameters	Correlation coefficient	*p* value
MD	0.44	0.01

Age	−0.09	0.63

IOP	−0.18	0.33

MAP	−0.19	0.31

Number of visual field tests	−0.14	0.47

Follow-up period	−0.24	0.20

PSV of the OA	−0.33	0.06
EDV of the OA	−0.26	0.16
RI of the OA	0.004	0.98

PSV of the CRA	0.37	0.04
EDV of the CRA	0.14	0.46
RI of the CRA	0.27	0.15

PSV of the NPCA	−0.20	0.29
EDV of the NPCA	0.13	0.51
RI of the NPCA	−0.43	0.01

PSV of the TPCA	−0.14	0.47
EDV of the TPCA	−0.14	0.48
RI of the TPCA	0.07	0.72

VFI: Visual Field Index.

MD: medial deviation.

IOP: intraocular pressure.

MAP: medial arterial pressure.

PSV: peak systolic velocity.

EDV: end-diastolic velocity.

RI: resistive index.

OA: ophthalmic artery.

CRA: central retinal artery.

NPCA: nasal posterior ciliary artery.

TPCA: temporal posterior ciliary artery.

**Table 3:** Compilation of studies focusing on correlations between CDI parameters and glaucoma progression.

Study	Study design	Patients	Methods	Follow-up	Significant findings related to visual field progression
Schumann et al. [17]	Retrospective	POAG (*n* = 20) with progressive visual field defects	VFI mean defect over time	50.1 ± 19.4 months	Mean blood flow velocities in the CRA, PSV, and RI in the OA

Gherghel et al. [18]	Retrospective	POAG (*n* = 20 with progressive visual field defects, *n* = 20 matched patients with stable disease)	Deepening of existing scotoma, expansion of existing scotoma, and fresh scotoma	5.3 ± 2.2 years	EDV in the CRA

Martínez and Sánchez [19]	Prospective	POAG (*n* = 49, 23 patients with progressive visual field defects)	Deepening of existing scotoma, expansion of existing scotoma, and fresh scotoma	36 months	RI in the OA and SPCAs

Satilmis et al. [20]	Retrospective	POAG (*n* = 20 with progressive visual field defects)	Deepening of existing scotoma, expansion of existing scotoma, fresh scotoma, and VFI mean defect over time	4.3 ± 1.6 years	EDV in the CRA

Galassi et al. [21]	Retrospective	POAG (*n* = 44, 18 with progressive visual field defects)	Progressive visual field defect recorded in at least 3 consecutive examinations	Up to 7 years	EDV and RI in the OA

Calvo et al. [22]	Prospective	Glaucoma suspects (*n* = 262, 36 with progressive disease)	Color coded Moorfields Regression Analysis (MRA) in confocal laser scanning system	48 months	RI > 0.75 in the CRA

Zeitz et al. [23]	Prospective	Glaucoma (*n* = 114, 12 with progressive visual field defects)	Increase in the cup-disc ratio of the optic disc and increase in MD	295 ± 18 days	PSV and EDV in the PCAs

Jimenez-Aragon et al. [24]	Prospective	POAG (*n* = 71, 12 with progressive visual field defects)	Color coded Moorfields Regression Analysis (MRA) in confocal laser scanning system	5 years	RI in the OA and CRA

Kuerten et al. [25]	Retrospective	NTG (*n* = 31)	VFI progression per year	7.6 ± 4.1 years	PSV of the CRA, RI of NPCA

NTG: normal tension glaucoma.

POAG: primary open-angle glaucoma.

VFI: Visual Field Index.

PSV: peak systolic velocity.

EDV: end-diastolic velocity.

RI: resistive index.

OA: ophthalmic artery.

CRA: central retinal artery.

NPCA: nasal posterior ciliary artery.

SPCA: small posterior ciliary artery.
